# Preparation of the Key Dolutegravir Intermediate via MgBr_2_-Promoted Cyclization

**DOI:** 10.3390/molecules26102850

**Published:** 2021-05-11

**Authors:** Jiahui Kong, Haijian Xia, Renbao He, Hao Chen, Yongping Yu

**Affiliations:** 1 Zhejiang Province Key Laboratory of Anti-Cancer Drug Research, College of Pharmaceutical Science, Zhejiang University, Hangzhou 310058, China; jiahui.kong@yongtaitech.com; 2 Zhejiang Yongtai Technology Co. Ltd., Taizhou 317016, China; haijian.xia@yongtaitech.com (H.X.); renbao.he@yongtaitech.com (R.H.); a_chenhaoHao@126.com (H.C.)

**Keywords:** dolutegravir, MgBr_2_-promoted cyclization, chemoselectivity

## Abstract

A novel approach for synthesizing the key dolutegravir intermediate is described via MgBr_2_-promoted intramolecular cyclization. Condensation of commercially available methyl oxalyl chloride and ethyl 3-(*N*,*N*-dimethylamino)acrylate afforded the vinylogous amide in an excellent yield. Subsequent substitution by aminoacetaldehyde dimethyl acetal and methyl bromoacetate gave rise to the expected precursor for cyclization, which was promoted by MgBr_2_ to highly selectively convert into pyridinone diester. The key dolutegravir intermediate was finally prepared by the selective hydrolysis of the corresponding diester via LiOH.

## 1. Introduction

**Scheme 1:**
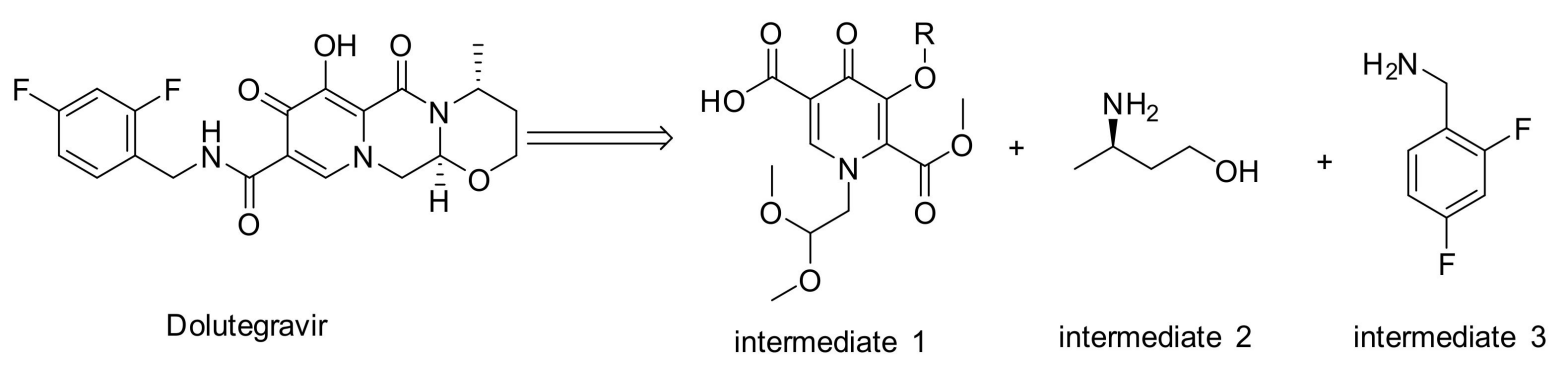
Chemical structures of dolutegravir and its intermediates.

HIV currently affects over 37 million people around the world [1,2,3]. In recent years, a number of innovative medicines have made HIV a manageable disease [4,5,6]. However, the cost of treatment is still prohibitive for many patients in lower-income countries. As a class of new anti-HIV drug, dolutegravir (DTG) developed by GlaxoSmithKline (GSK) and Shionogi exhibits outstanding potent antiviral activity and high genetic barrier to resistance and has been used worldwide since its launch into the market in 2013 (Scheme 1).

**Scheme 2:**
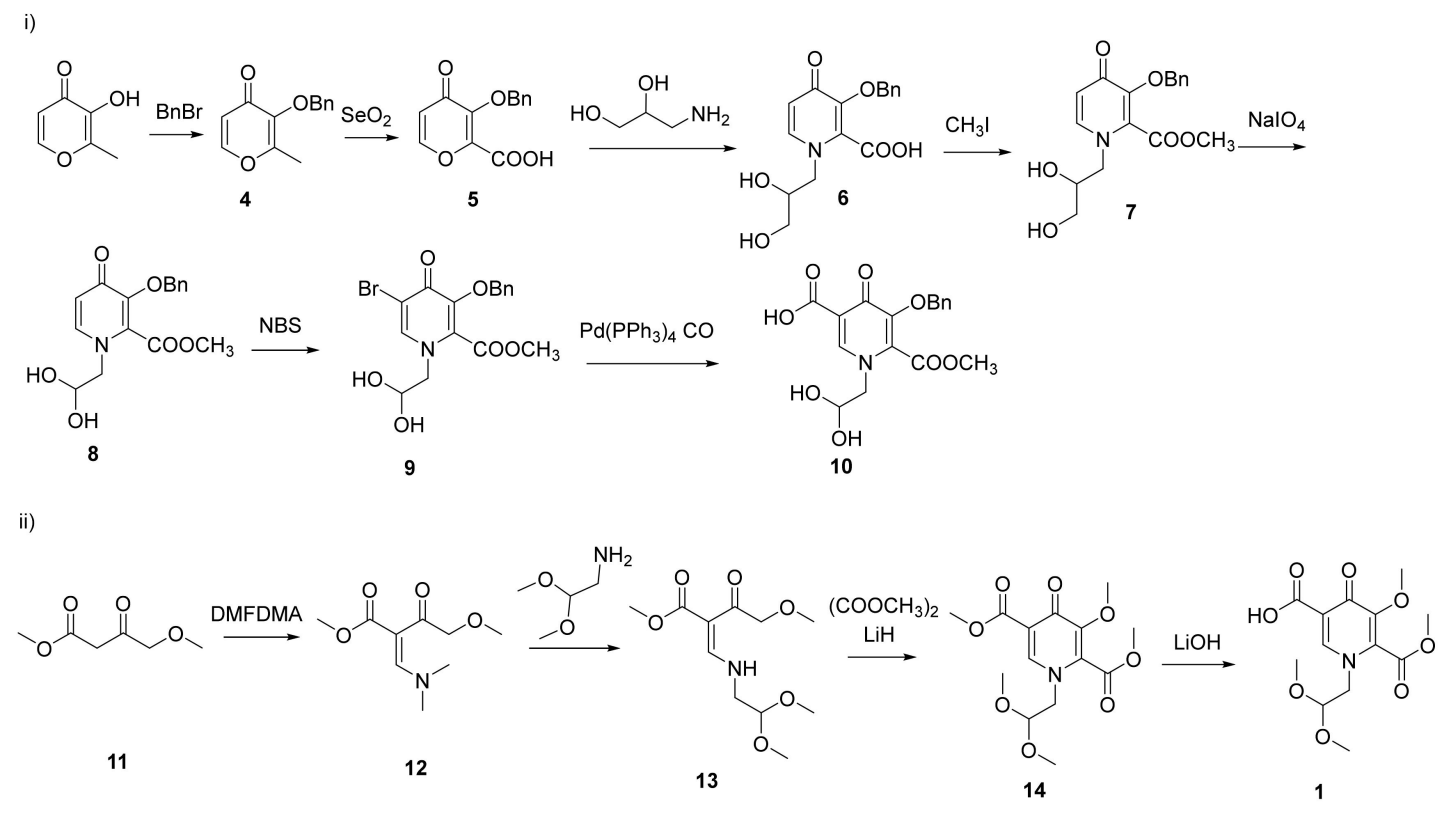
Previous synthetic routes of intermediate **1**.

The synthesis of dolutegravir was firstly reported by GSK [7]. At present, most of the synthetic routes for dolutegravir involve three key intermediates: pyridinone moiety (intermediate **1**), (R)-3-aminobutan-1-ol (intermediate **2**) and (2,4-difluorophenyl)methanamine (intermediate **3**) (Scheme 1). The pyridinone moiety is a common building block in pharmaceutically active compounds. Obviously, intermediate **1** is the core unit and plays the leading role in synthesizing dolutegravir. Up to date, there were mainly two synthetic routes for the synthesis of intermediate **1**. The first route (Scheme 2i) took maltol as the starting material [8,9,10]. Protected maltol (**4**) was oxidized by SeO_2_ to afford maltol α-acid (**5**), which was substituted by 3-aminopropane-1,2-diol to afford pyridinone (**6**). The ester (**7**) was obtained by treating **6** with iodomethane. Subsequent oxidation by NaIO_4_ and bromination with NBS afforded the key intermediate (**9**). Finally, carboxylated catalyzed by Pd(PPh_3_)_4_, pyridinone intermediate **10** (similar structure with intermediate **1**) was synthesized in a low yield. This route required numerous synthetic steps along with tedious chromatographic purification processes, leading to a low total yield (5∼10%). Moreover, other drawbacks of this route include the severe pollutions, the harsh condition of oxidation and the unacceptable cost resulting from SeO_2_, CH_3_I, NaIO_4_ and Pd(PPh_3_)_4_. In recent years, a new generation of synthetic route (Scheme 2ii) has aroused intensive interest, which started from substituted ethyl acetoacetate [11,12,13,14,15,16,17,18]. 4-methoxyacetoacetic acid methyl ester(**11**) was synthesized by chlorination of diketene and subsequent substitution via NaH in MeOH/THF [19,20]. Treating **11** with *N*,*N*-dimethylformamide dimethyl acetal (DMFDMA) afforded the vinylogous amide (**12**) in a 80~85% yield. Further substitution by aminoacetaldehyde dimethyl acetal, intermolecular ring closure promoted by LiH and selective hydrolysis, the intermediate **1** was synthesized in a 62% total yield from **11**. Compared with route 1, the total synthetic steps of route 2 were greatly decreased which was beneficial to industrial application. However, the starting material (**11**) was expensive and its syntheses involved harsh conditions and dangerous materials (diketene and chlorine). Furthermore, the ring-closure step involved dangerous reagents such as LiH or NaH.

**Scheme 3:**
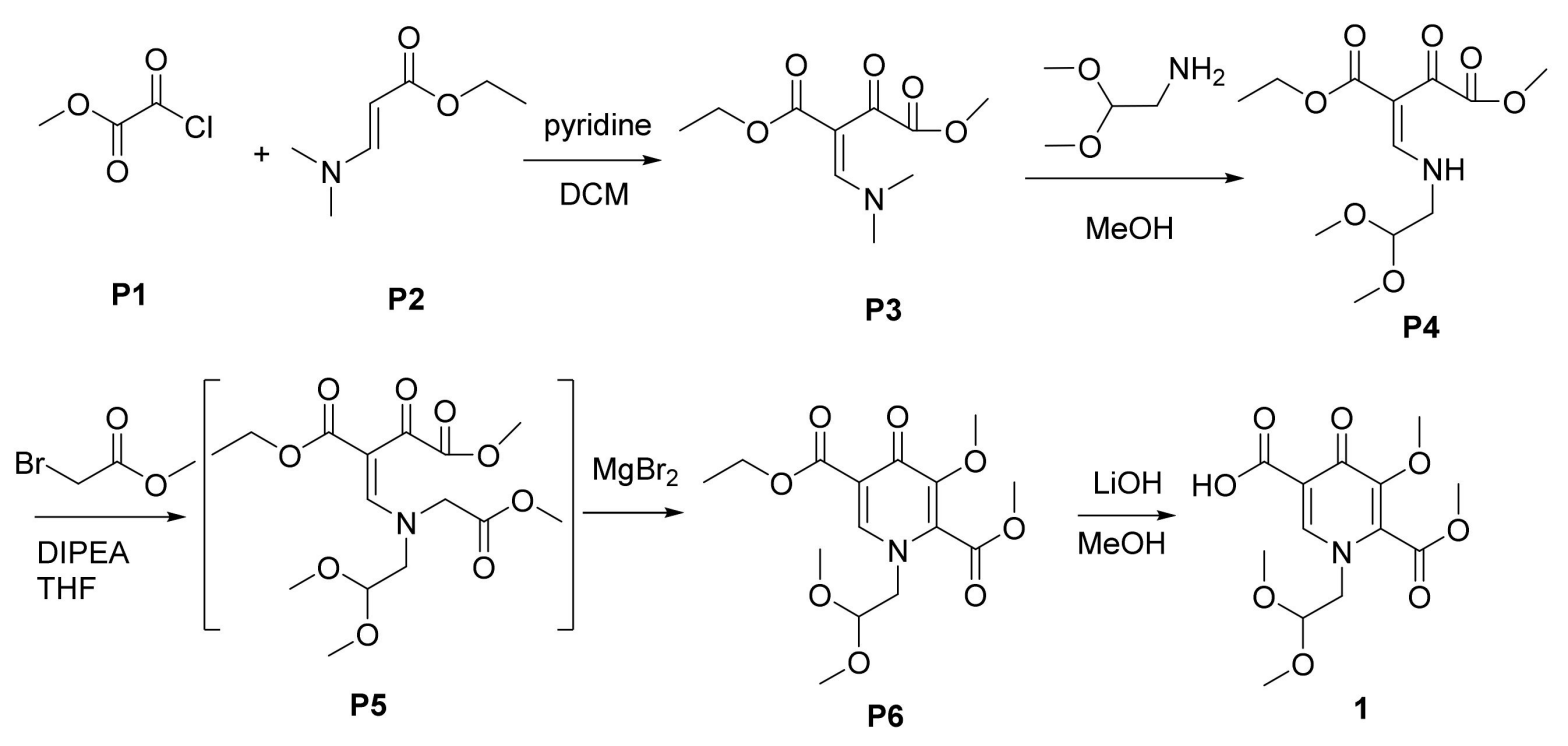
Novel syntheses of intermediate **1**.

As aforementioned, the syntheses of intermediate **1** are facing the problems of low yields, high cost and harsh conditions. Therefore, for industrial production, it urgently needs to improve the processes. Herein, we report an important improvement of the key ring-closure condition for practical scale production of the dolutegravir intermediate **1** which involves the inexpensive materials and moderate reaction conditions compared with previous routes (Scheme 3). To our best of knowledge, prior to this work, there has been no report for the direct formation of pyridinones via MgBr_2_-promoted cyclization.

## 2. Results and Discussion

The synthetic routes are depicted in Scheme 3. In designing a more efficient and robust synthetic route of intermediate **1**, it was initially envisioned that the central pyridinone ring could be derived from **P5**, which in turn could be obtained by reacting a vinylogous amide **P4** with methyl bromoacetate [21,22,23]. There are two main challenges in the chemical synthesis of **1**: the cyclization of **P5** and the selective hydrolysis of **P6**.

Treatment of **P1** (1.0 equiv) and **P2** (1.0 equiv) with pyridine (1.2 equiv) in DCM furnished the corresponding product **P3** cleanly. Pyridine was proved to be favorable base for this step. Compared with pyridine, trimethylamine (TEA) or sodium methoxide decreased the yields obviously (Table 1, entry 1, 5). Moreover, the environmentally-friendly base, magnesium methoxide, displayed the acceptable result with the yield up to 85% (Table 1, entry 6). However, the byproduct (dimethyl oxalate) had been detected from the reaction mixture which resulted in ~20% **P2** remained. Accordingly, the amount of **P1** also should be increased to 1.3 equiv in order to consume **P2** fully. Temperature optimization experiments (Table 1, entry 3, 4) indicated that the lower temperature (<−5 °C) hindered the reaction and the side reactions appeared upon the raised temperature (>5 °C). The pure **P3** was obtained in a 95% yield after refining.

**Scheme 4:**
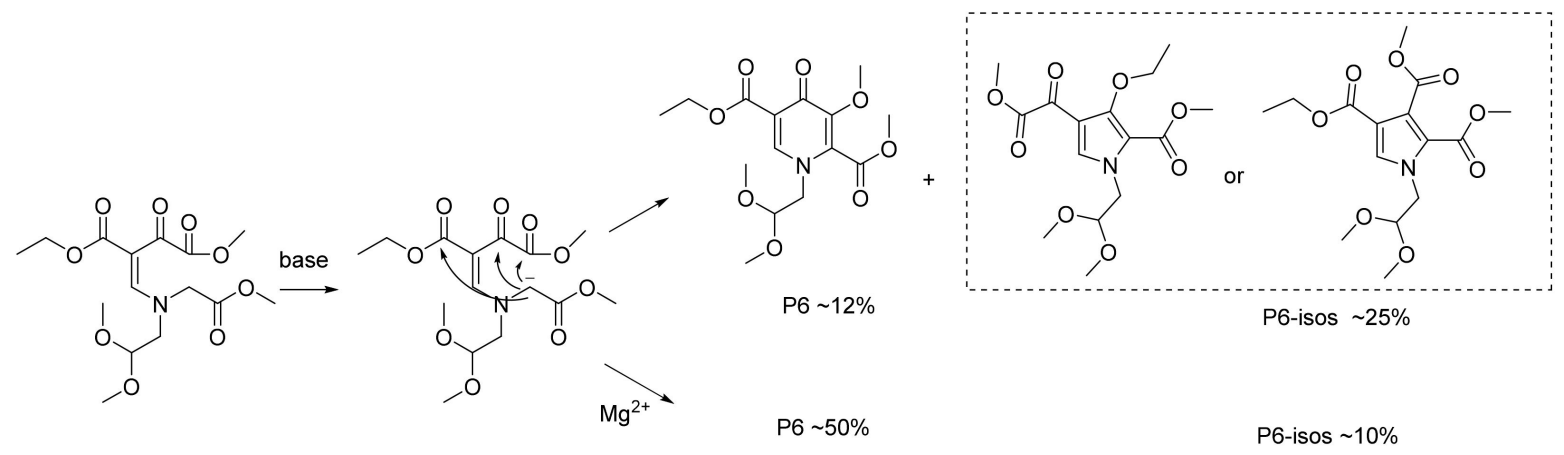
The possible mechanism of **P6** and **P6-isos**.

With **P3** in hand, **P4** was prepared via the condensation of **P3** with aminoacetaldehyde dimethyl acetal. The further substitution with methyl bromoacetate successfully converted **P4** into **P5**. When the reaction was proceeded with the base of trimethylamine (Table 1, entry 1), the desired product (**P6**) was detected in a very low yield (~12%), while the possible isomers **P6-isos** (~25%) and other byproducts (which were possible to be polymers or degradation products according to LCMS) appeared obvious. These possible isomers were speculated to be formed through another styles of cyclization under the basic condition (Scheme 4) according to the similar molecular weight in LCMS (Figure S13 in Supplementary Material). To improve the chemoselectivity, the reaction conditions were extensively explored.

We first examined the factor of base for forming the more reactive carbanion. The results revealed that organic base (trimethylamine and *N*,*N*-diisopropylethylamin) with the better solubility in THF was more inclined to promote the reaction (Table 2, entry 1, 2). *N*,*N*-diisopropylethylamine presented a more suitable candidate compared with trimethylamine. On the other hand, sodium *tert*-butoxide generated the decomposition of substrate (Table 2, entry 3). Considering that magnesium ions usually played an important role in cyclization [24], we next studied the effect of Mg^2+^. Surprisingly, when MgBr_2_ (0.1 equiv relative to the substrate) was added to the reaction mixture, the yield of desired product **P6** increased astoundingly with the contents of **P6-isos** decreased obviously, which revealed the chemoselectivity were successfully influenced by MgBr_2_ (Table 2, entry 4). Moreover, the effect various magnesium salts were researched (Table 2, entry 5~8). Compared with MgBr_2_, MgCl_2_ and MgSO_4_ indicated the grudgingly acceptable results. The more expensive magnesium salt, MgI_2_, revealed a slightly increased yield, while Mg(OCH_3_)_2_ (0.5 equiv) without additional base and additive displayed the unsatisfactory result due to the decomposition of substrate. Considering the cost of MgBr_2_ and MgI_2_, the advantages of inexpensive MgBr_2_ were apparent.

**Scheme 5:**
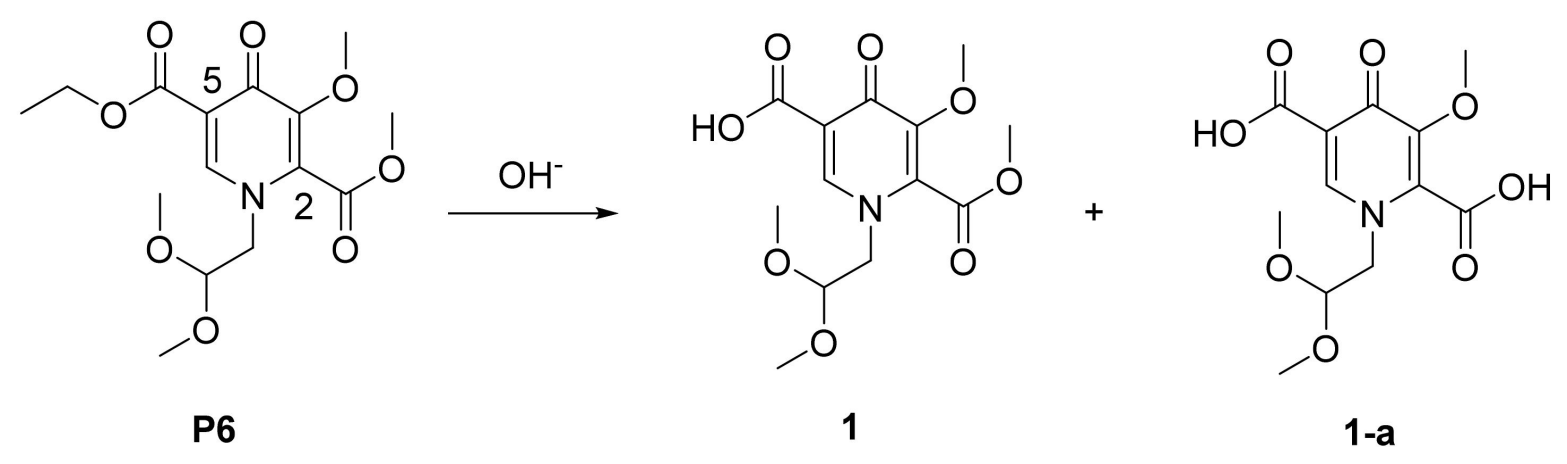
The selective hydrolysis of **P6**.

Finally, the hydrolysis of **P6** proved challenging. Competitive hydrolysis of the C-2 ester took place under a variety of conditions (Scheme 5). The inexpensive inorganic base such as NaOH or KOH revealed a low selectivity with the content of byproduct diacid (**1-a**) unacceptable (Table 3, entry 1, 2). Interestingly, LiOH presented a satisfactory selectivity contributed to its soft base nature (Table 3, entry 3). Moreover, hydrolysis of **P6** seemed temperature sensitive. High temperature damaged the high chemoselectivity (Table 3, entry 5). Understandably, low temperature was contributed to improve chemoselectivity slightly along with the slowed reaction speed obviously (Table 3, entry 4).

## 3. Materials and Methods

### 3.1. Materials and Instrumentation

Commercially available solvents and reagents were used without further purification unless otherwise mentioned. Thin-layer chromatography (TLC) was carried out on aluminum sheets coated with silica gel 60 F254 (MERCK, Darmstadt, Germany). ^1^H NMR spectra were obtained using a Bruker AM 400 spectrometer (Leipzig, Germany), and the chemical shifts were reported relative to tetramethylsilane (*δ* = 0) in ppm. ^13^C NMR spectra were recorded at 100 MHz (Bruker AM 400, Leipzig, Germany) and the chemical shifts were reported relative to CDCl_3_ (*δ* = 77.00) or DMSO-*d_6_* (*δ* = 39.52) in ppm. The full characterization spectra of **P3**, **P4**, **P6** and **1** were presented in Figures S1–S12.

### 3.2. Syntheses

#### 3.2.1. Synthesis of **P3**

To a stirred solution of (143 g, 1.0 mol) ethyl 3-(*N*,*N*-dimethylamino)acrylate and (95 g, 1.2 mol) pyridine in DCM (500 mL) was added the DCM solution of (122 g, 1.0 mol) methyl oxalyl chloride under nitrogen with the temperature below 5 °C. The reaction mixture was kept at 5 °C for 20 min. Then, the mixture was allowed to warm to room temperature. After 2 h, the reaction was quenched with 200 mL 5% NaHCO_3_ aqueous solution. The organic phase was separated and washed with 100 mL water. The solvent was evaporated in vacuo. The crude product was dissolved in 654 g methyl *tert*-butyl ether (MTBE) and heated to reflux. The mixture was slowly cooled to 50 °C. The seed crystal (1 g) was added and stirred for 1 h, then slowly cooled to −5 °C. After filtration, the filter cake was washed with cold MTBE (100 mL) and the filter cake was dried to afford **P3** (218 g, 95% yield). ^1^H NMR (400 MHz, CDCl_3_) δ 7.86 (s, 1H), 4.17 (d, *J* = 7.1 Hz, 2H), 3.84 (s, 3H), 3.37 (s, 3H), 3.04 (s, 3H), 1.27 (t, *J* = 7.1 Hz, 3H). ^13^C NMR (100 MHz, CDCl_3_) δ 183.0, 166.6, 166.4, 160.1, 97.4, 60.8, 52.1, 48.4, 43.2, 14.2. LC-MS: *m*/*z*; [M + H]^+^, calcd for C_10_H_16_NO_5_: 230.10; found: 230.16.

#### 3.2.2. Synthesis of **P4**

To a stirred solution of (229 g, 1.0 mol) **P3** in MeOH (500 mL) was added (110 g, 1.05 mol) aminoacetaldehyde dimethyl acetal with the temperature below 15 °C. The reaction mixture was kept at 15 °C for 30 min. Then, the solvent was evaporated in vacuo. The crude product was dissolved in 600 g methyl *tert*-butyl ether (MTBE) and 400 g *n*-hexane, and heated to reflux. The mixture was slowly cooled to −5 °C. After filtration, the filter cake was washed with cold *n*-hexane (100 mL) and dried to afford **P4** (262 g, 90% yield). ^1^H NMR (400 MHz, CDCl3) δ 10.62 (s, 1H), 8.05 (d, *J* = 14.1 Hz, 1H), 4.45 (dd, *J* = 4.2, 2.9 Hz, 1H), 4.25–4.12 (m, 2H), 3.95–3.78 (m, 3H), 3.57–3.49 (m, 2H), 3.47–3.34 (m, 6H), 1.39–1.15 (m, 3H). ^13^C NMR (100 MHz, CDCl_3_) δ 186.3, 165.9, 165.7, 161.2, 102.5, 60.2, 55.1, 55.0, 52.1, 14.2. LC-MS: *m*/*z*; [M + H]^+^, calcd for C_12_H_20_NO_7_: 290.12; found: 290.18.

#### 3.2.3. Synthesis of **P6**

To the solution of **P4** (145 g, 0.50 mol) in THF (400 mL) was added (65 g, 0.50 mol) *N*,*N*-Diisopropylethylamine (DIPEA) under nitrogen with the temperature below −5 °C. The resulting solution was stirred at −5 °C for 30 min. A solution of methyl bromoacetate (84 g, 0.50 mol) in pre-dried THF (150 mL) was added over 30 min and the reaction mixture was kept at −5 °C for 30 min. Then, MgBr_2_ (10 g, 55 mmol) was added and the mixture was allowed to warm to room temperature. After 2 h, the reaction was quenched with 100 mL saturated NH_4_Cl aqueous solution. The organic phase (containing ~55% **P6**, ~10% **P6-isos** and ~35% other byproducts) was separated and immediately used without further purification. The pure **P6** was obtained by flash column chromatography (PE:EA = 1:1 to 1:3). ^1^H NMR (500 MHz, CDCl3) δ 8.14 (s, 1H), 4.52 (t, *J* = 4.8 Hz, 1H), 4.35 (q, *J* = 7.1 Hz, 2H), 4.02–3.95 (m, 8H), 3.41 (s, 6H), 1.37 (t, *J* = 7.1 Hz, 3H). ^13^C NMR (100 MHz, CDCl_3_) δ 170.9, 164.4, 162.3, 150.0, 145.7, 133.8, 118.5, 102.7, 60.9, 60.4, 56.5, 55.7, 53.2, 14.2. LC-MS: *m*/*z*; [M + H]^+^, calcd for C_15_H_22_NO_8_: 344.13; found: 343.86

#### 3.2.4. Synthesis of **1**

The organic phase containing **P6** was added 100 g water. The mixture was cooled to 0 °C. LiOH·H_2_O (41 g, 1.0 mol) was added to the mixture in three portions with the temperature below 0 °C. The reaction mixture was kept at 0 °C for 6 h. The reaction mixture was stirred and monitored by TLC. After complete hydrolysis of **P6,** the reaction was quenched by 1M HCl (~300 mL) and was extracted with CH_2_Cl_2_. The organic layer was washed with 5% aqueous sodium hydrogen carbonate (100 mL) and 2% aqueous sodium chloride (100 mL). After removal of the solvent, the crude was dissolved in isopropanol (500 mL) by heating. The solution was gradually cooled to −5 °C. Filtration, washing with isopropanol (50 mL), and drying provided pure **1** (50 g, 45% yield, HPLC purity 99.9%). ^1^H NMR (400 MHz, CDCl3) δ 15.04 (s, 1H), 8.47 (s, 1H), 4.56 (t, *J* = 4.5 Hz, 1H), 4.19 (t, *J* = 7.1 Hz, 2H), 4.02 (d, *J* = 4.2 Hz, 6H), 3.42 (s, 6H). ^13^C NMR (100 MHz, CDCl_3_) δ 174.8, 161.5, 148.6, 145.4, 136.6, 116.5, 102.3, 60.9, 57.2, 55.9, 53.7. LC-MS: *m*/*z*; [M + H]^+^, calcd for C_13_H_18_NO_8_: 316.10; found: 316.17.

## 4. Conclusions

In summary, we herein report a novel and efficient synthetic method for the dolutegravir intermediate. By condensation of methyl oxalyl chloride (**P1**) and ethyl 3-(*N*,*N*-dimethylamino)acrylate (**P2**), **P3** was obtained in an excellent yield. The subsequent substitution with aminoacetaldehyde dimethyl acetal afforded **P4** in near equivalent yield. The further condensation of methyl bromoacetate and **P4** afforded the intermediate **P5** which was directly used for the synthesis of **P6**. Surprisingly, promoted by MgBr_2_, intramolecular cyclization of **P5** proceeded smoothly. Fortunately, by treating **P6** with LiOH, the expected highly selective hydrolysis product **1** was successfully synthesized. In conclusion, these results compose a convenient approach for the synthesis of the key dolutegravir intermediate. Furthermore, optimizing the reaction condition of **P5** and increasing the yield will be our next major project.

## Figures and Tables

**Table 1:** Base and temperature effects on the syntheses of **P3.**

Entry	Base	Temperature	Yield
1	TEA (1.2 equiv)	−5 °C	56%
2	Pyridine (1.2 equiv)	−5 °C	95%
3	Pyridine (1.2 equiv)	−10 °C	90%
4	Pyridine (1.2 equiv)	8 °C	79%
5	NaOCH_3_ (1.2 equiv)	−5 °C	36%
6	Mg(OCH_3_)_2_ (0.6 equiv)	−5 °C	85%

TEA: trimethylamine.

**Table 2:** Base and additive effects on the syntheses of **P6.**

Entry	Base	Additive	Yield ^a^	
			P6	P6-Isos
1	TEA (0.5 equiv)	\	10%	25%
2	DIPEA (1.0 equiv)	\	20%	25%
3	sodium tert-butoxide (1.0 equiv)	\	2%	2%
4	DIPEA (1.0 equiv)	MgBr_2_	50%	10%
5	DIPEA (1.0 equiv)	MgCl_2_	38%	15%
6	DIPEA (1.0 equiv)	MgI_2_	51%	10%
7	DIPEA (1.0 equiv)	MgSO_4_	23%	20%
8	Mg(OCH_3_)_2_ (0.5 equiv)	\	10%	10%

DIPEA: *N*,*N*-diisopropylethylamin. ^a^ The yields of **P6** and **P6-isos** were analyzed based on TLC or HPLC results.

**Table 3:** Base effect on the syntheses of **1.**

Entry	Base	Temperature	Yield	
			1	1-a
1	NaOH (2.0 equiv)	0 °C	62%	32%
2	KOH (2.0 equiv)	0 °C	59%	35%
3	LiOH (2.0 equiv)	0 °C	90%	8%
4 *	LiOH (2.0 equiv)	−10 °C	91%	7%
5	LiOH (2.0 equiv)	10 °C	82%	15%

* the reaction time was 24 h, and others were 6 h.

## Data Availability

The data presented in this study are available on request from the corresponding author.

## Supplementary Materials

Supplementary materials are available online. Figure S1: ^1^HNMR spectra of **P3** in CDCl_3_. Figure S2: ^13^CNMR spectra of **P3** in CDCl_3_. Figure S3: LCMS of **P3**. Figure S4: ^1^HNMR spectra of **P4** in CDCl_3_. Figure S5: ^13^CNMR spectra of **P4** in CDCl_3_. Figure S6: LCMS of **P4**. Figure S7: ^1^HNMR spectra of **P6** in CDCl_3_. Figure S8: ^13^CNMR spectra of **P6** in CDCl_3_. Figure S9: LCMS of **P6**. Figure S10: ^1^HNMR spectra of **1** in CDCl_3_. Figure S11: ^13^CNMR spectra of **1** in CDCl_3_. Figure S12: LCMS of **1**. Figure S13: LCMS of **P6-isos.**

## Sample Availability

Samples of the compounds are not available from the authors.
