# Supplementary material for: Preparation of the Key Dolutegravir Intermediate via MgBr2-Promoted Cyclization
Source: Molecules. 2021 May 11;26(10):2850. doi: 10.3390/molecules26102850 (PMC8150840; doi:10.3390/molecules26102850)
Supplement: Supplementary file 1 [file molecules-26-02850-s001.zip › molecules-1202555-supplementary.pdf]

Supporting Information

Preparation of the Key Dolutegravir Intermediate via $MgBr_2$-Promoted Cyclization

Jiahui Kong [1,2], Haijian Xia [2], Renbao He [2], Hao Chen [2] and Yongping Yu [1,*]

[1] Zhejiang Province Key Laboratory of Anti-Cancer Drug Research, College of Pharmaceutical Science, Zhejiang University, Hangzhou 310058, China; jiahui.kong@yongtaitech.com (J.K.)
[2] Zhejiang Yongtai Technology Co. Ltd., Taizhou 317016, China; haijian.xia@yongtaitech.com (H.X.); renbao.he@yongtaitech.com (R.H.); a_chenhaoHao@126.com (H.C.)
* Correspondence: yyu@zju.edu.cn

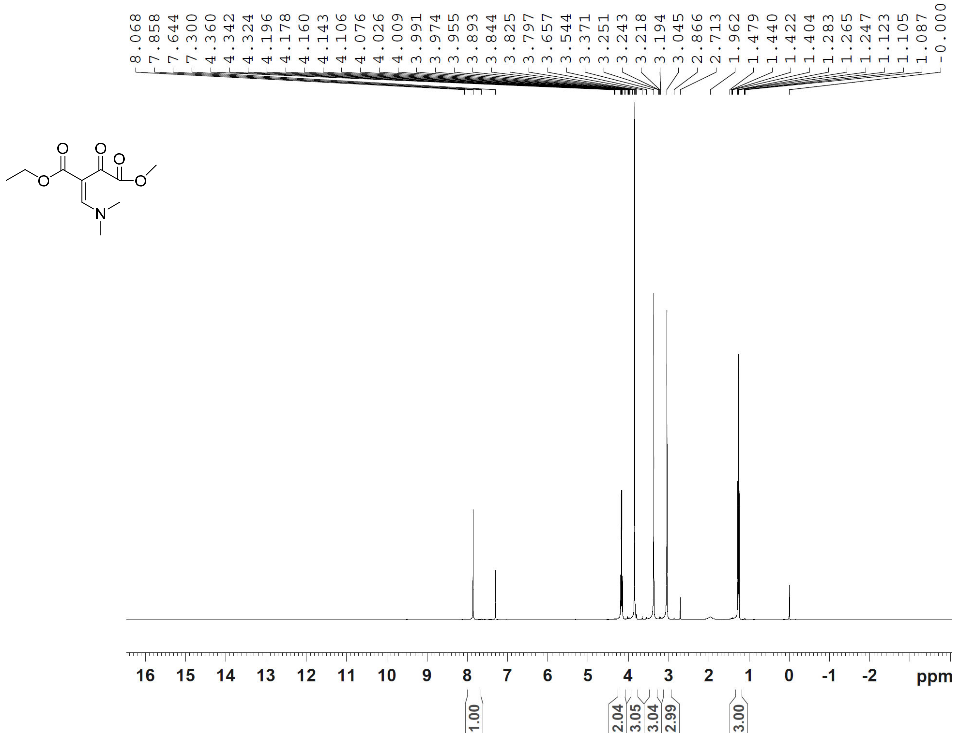

Figure S1. ^{1}HNMR spectra of **P3** in $CDCl_3$.

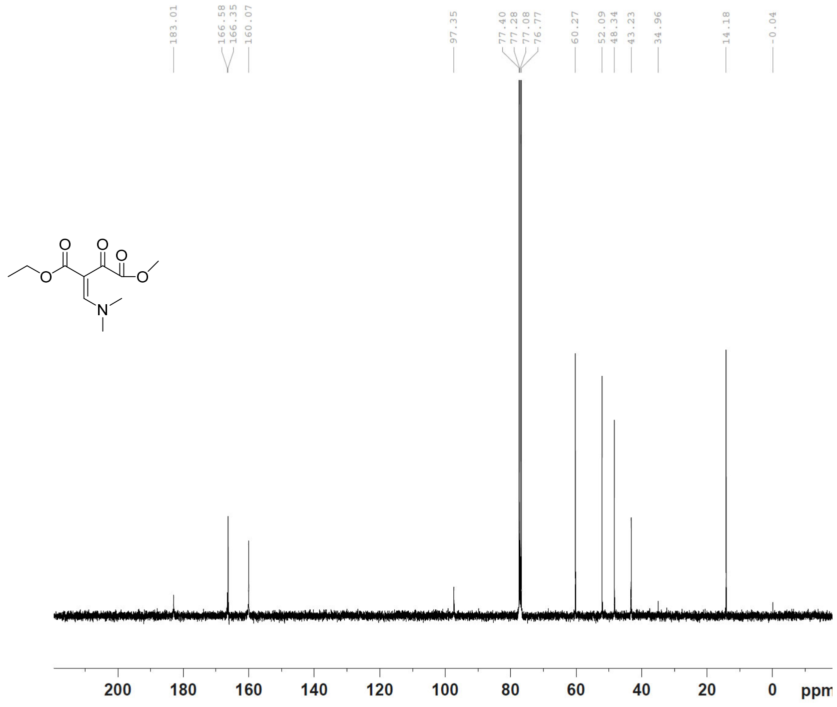

Figure S2. ^{13}CNMR spectra of **P3** in $CDCl_3$.

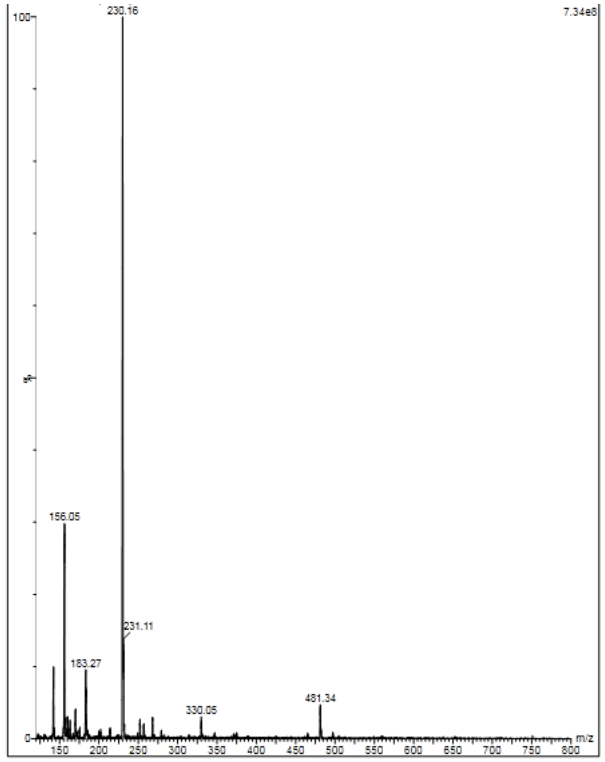

Figure S3. LCMS of **P3.**

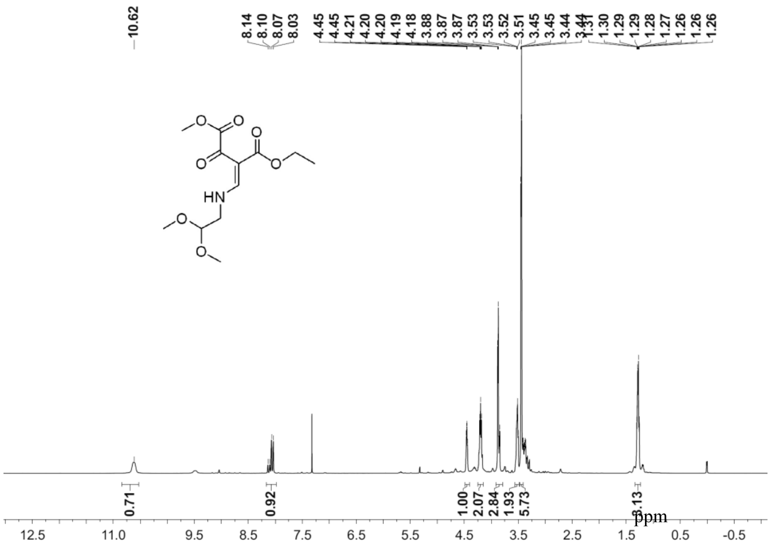

Figure S4. ^{1}HNMR spectra of **P4** in $CDCl_3$.

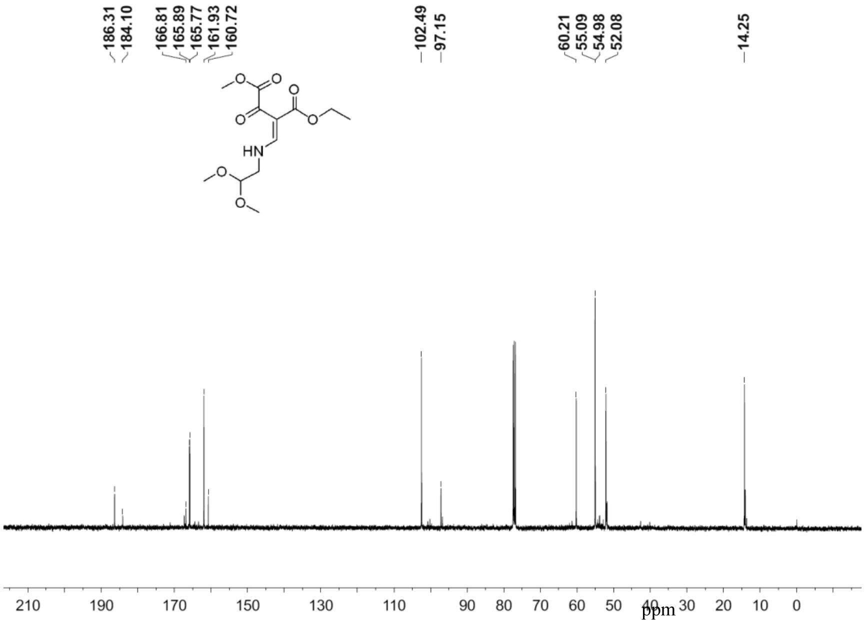

Figure S5. ^{13}CNMR spectra of **P4** in $CDCl_3$.

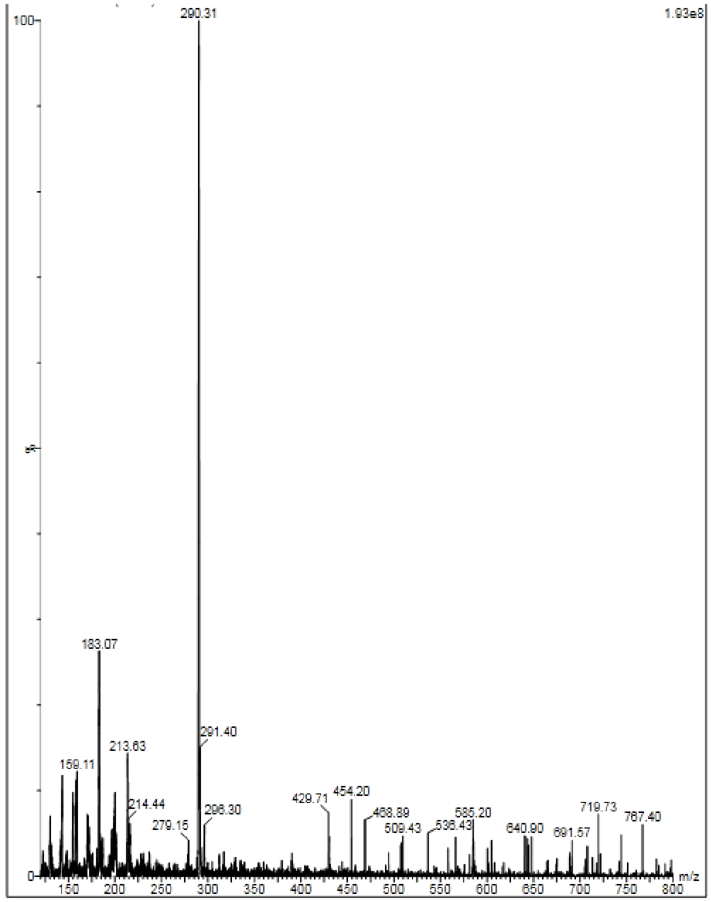

Figure S6. LCMS of **P4.**

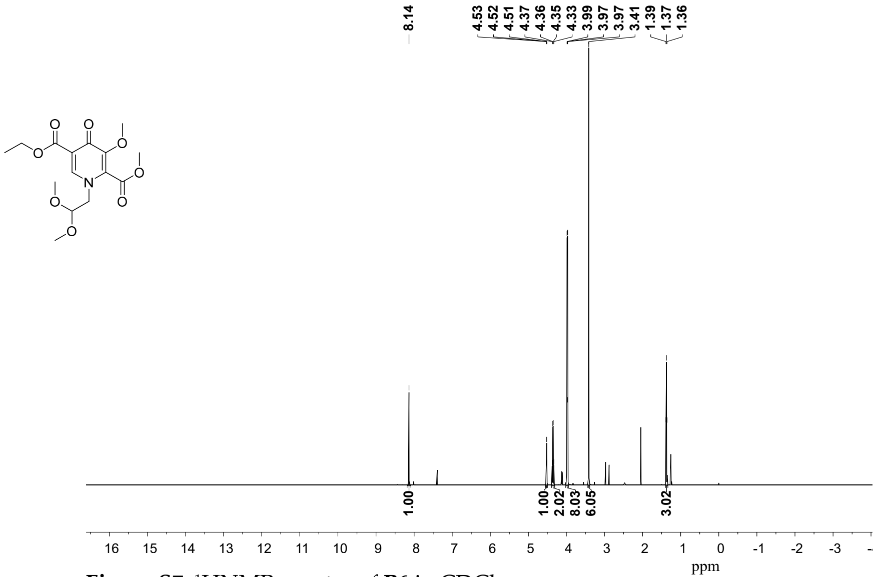

Figure S7. ^{1}HNMR spectra of **P6** in $CDCl_3$.

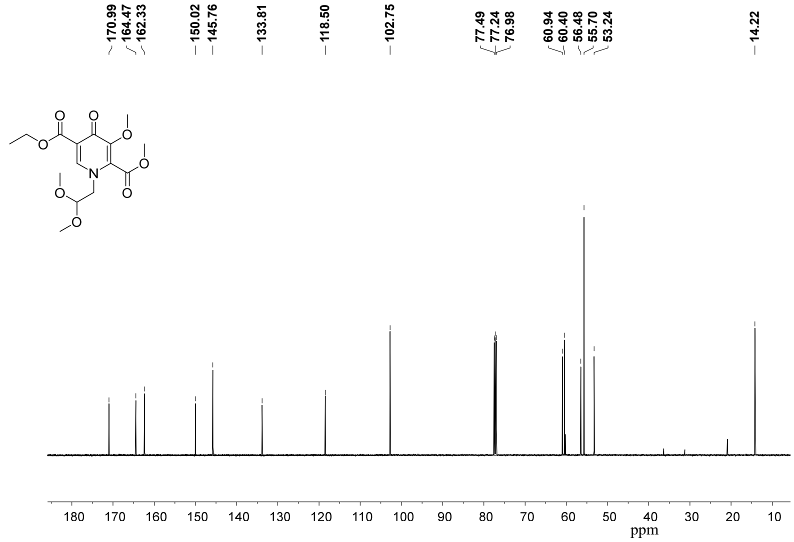

Figure S8. ^{13}CNMR spectra of **P6** in $CDCl_3$.

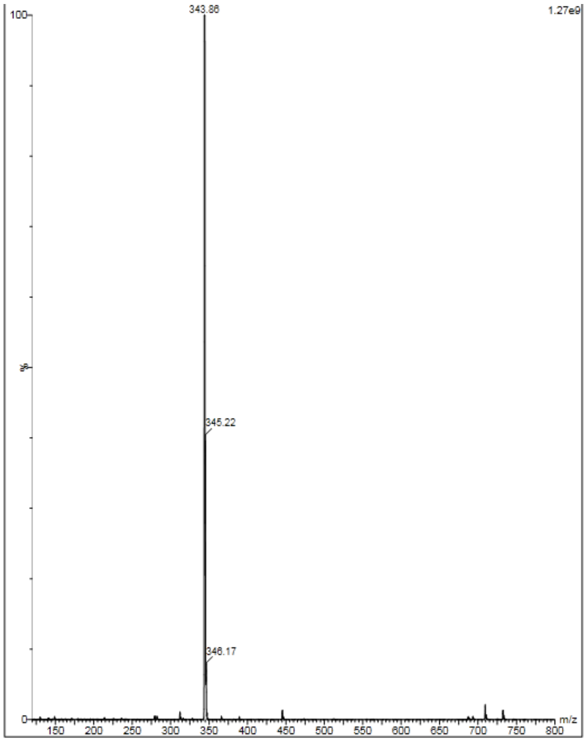

Figure S9. LCMS of **P6.**

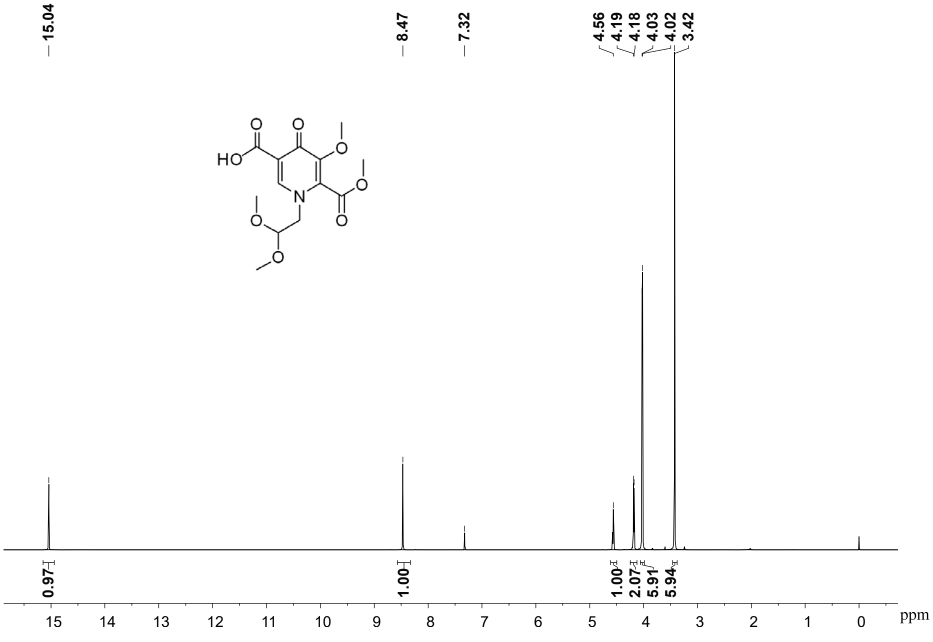

Figure S10. ¹HNMR spectra of **1** in $CDCl_3$.

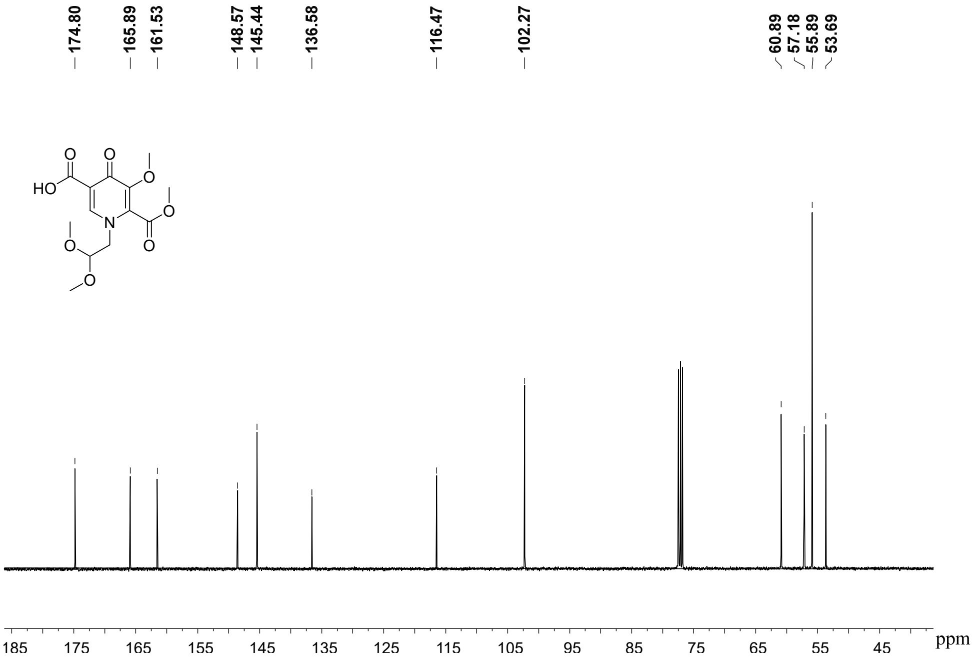

Figure S11. ^{13}CNMR spectra of **1** in $CDCl_3$.

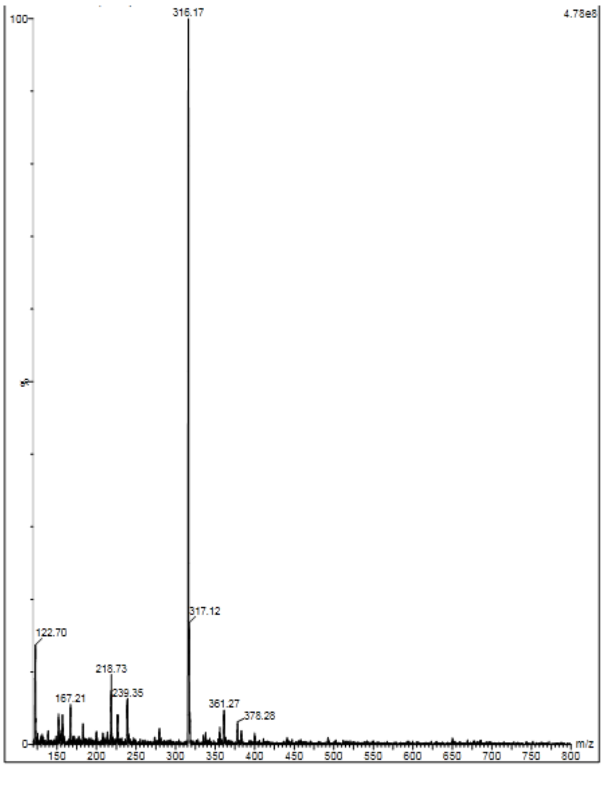

Figure S12. LCMS of **1.**

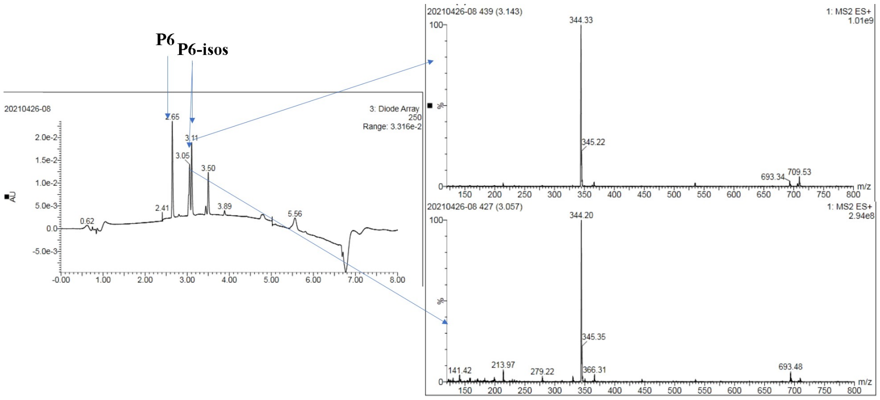

Figure S13. LCMS of **P6-isos**
